# (1*E*)-1-(3-Bromo­phen­yl)ethanone 2,4-di­nitro­phenyl­hydrazone

**DOI:** 10.1107/S1600536810037980

**Published:** 2010-10-20

**Authors:** Jerry P. Jasinski, Curtis J. Guild, C. S. Chidan Kumar, H. S. Yathirajan, A. N. Mayekar

**Affiliations:** a Department of Chemistry, Keene State College, 229 Main Street, Keene, NH 03435-2001, USA; b Department of Studies in Chemistry, University of Mysore, Manasagangotri, Mysore 570 006, India; c Department of Studies in Chemistry, University of Mysore, Manasagangotri, Mysore 570 006, India, and, SeQuent Scientific Ltd, Baikampady, New Mangalore 575 011, India

## Abstract

The title compound, C_14_H_11_BrN_4_O_4_, contains 3-bromo­phenyl and 2,4-dinitro­phenyl groups on opposite sides of a hydrazone unit and crystallizes with two mol­ecules in the asymmetric unit. The dihedral angles between the two ring systems in each mol­ecule are 2.0 (1) and 2.5 (4)°. Weak C—H⋯O hydrogen bonds and weak π–π stacking inter­actions [centroid–centroid distance = 3.7269 (14) Å] help to establish the packing. Intra­molecular N—H⋯O hydrogen bonds are also observed. On one of the rings, the Br atom is disordered over two equivalent positions of the phenyl ring [occupancy ratio 0.8734 (10):0.1266 (10).

## Related literature

For background to Schiff bases and their complexes, see: Baughman *et al.* (2004 ▶); El-Seify *et al.* (2006 ▶); Liang *et al.* (2007 ▶); Okabe *et al.* (1993 ▶); Zare *et al.* (2005 ▶). For related structures, see: Bolte & Dill, (1998 ▶); Fan *et al.* (2004 ▶); Ji *et al.* (2010 ▶); Kia *et al.* (2009 ▶); Motherwell & Ramsay, (2007 ▶); Shan *et al.* (2002 ▶). For bond-length data, see: Allen *et al.* (1987 ▶).
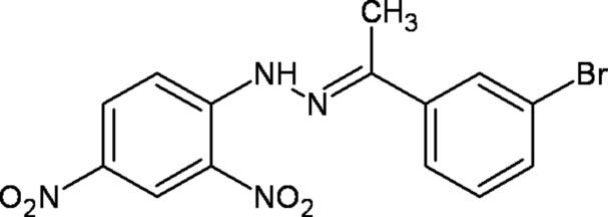


## Experimental

### 

#### Crystal data

C_14_H_11_BrN_4_O_4_
*M* *_r_* = 379.18
Triclinic, 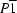

*a* = 7.7546 (9) Å
*b* = 13.4362 (15) Å
*c* = 14.1884 (16) Å
α = 91.894 (2)°
β = 90.553 (2)°
γ = 97.128 (2)°
*V* = 1466.0 (3) Å^3^
*Z* = 4
Mo *K*α radiation
μ = 2.83 mm^−1^
*T* = 100 K
0.55 × 0.55 × 0.24 mm

#### Data collection

Bruker APEXII CCD diffractometer
Absorption correction: multi-scan (*SADABS*; Bruker, 2008 ▶) *T*_min_ = 0.547, *T*_max_ = 0.746
16795 measured reflections
8581 independent reflections
6680 reflections with *I* > 2σ(*I*)
*R*_int_ = 0.024

#### Refinement

*R*[*F* ^2^ > 2σ(*F* ^2^)] = 0.040
*wR*(*F* ^2^) = 0.102
*S* = 1.04
8581 reflections
428 parameters
2 restraints
H-atom parameters constrained
Δρ_max_ = 1.44 e Å^−3^
Δρ_min_ = −0.77 e Å^−3^

### 

Data collection: *APEX2* (Bruker, 2008 ▶); cell refinement: *SAINT* (Bruker, 2008 ▶); data reduction: *SAINT*; program(s) used to solve structure: *SHELXS97* (Sheldrick, 2008 ▶); program(s) used to refine structure: *SHELXL97* (Sheldrick, 2008 ▶); molecular graphics: *SHELXTL* (Sheldrick, 2008 ▶); software used to prepare material for publication: *SHELXTL*.

## Supplementary Material

Crystal structure: contains datablocks global, I. DOI: 10.1107/S1600536810037980/bv2160sup1.cif

Structure factors: contains datablocks I. DOI: 10.1107/S1600536810037980/bv2160Isup2.hkl

Additional supplementary materials:  crystallographic information; 3D view; checkCIF report

## Figures and Tables

**Table 1:** Hydrogen-bond geometry (Å, °)

*D*—H⋯*A*	*D*—H	H⋯*A*	*D*⋯*A*	*D*—H⋯*A*
N1*B*—H1*B*⋯O1*B*	0.86	1.99	2.615 (2)	129
N1*A*—H1*A*⋯O1*A*	0.86	2.00	2.621 (2)	128
C8*B*—H7⋯O2*A*^i^	0.98	2.60	3.241 (3)	123
C12*B*—H12*B*⋯O3*B*^i^	0.95	2.38	3.332 (3)	175
C8*A*—H8*A*⋯O3*B*^ii^	0.98	2.61	3.369 (3)	135
C13*A*—H13*A*⋯O4*B*^iii^	0.95	2.40	3.282 (3)	154

Symmetry codes: (i) 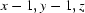; (ii) 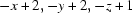; (iii) 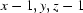.

**Fig. 1.**
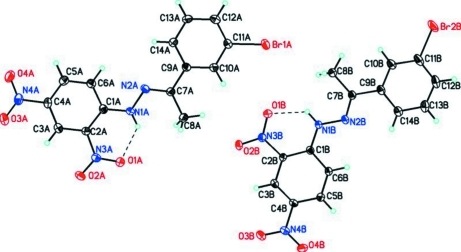
Molecular structure of C14H11N4O4Br, showing the atom labeling scheme with 50% probability displacement ellipsoids with two molecules in the asymmetric unit. In molecule B only the major component [Br2B (0.873 (1) occupancy]) is depicted.

**Fig. 2.**
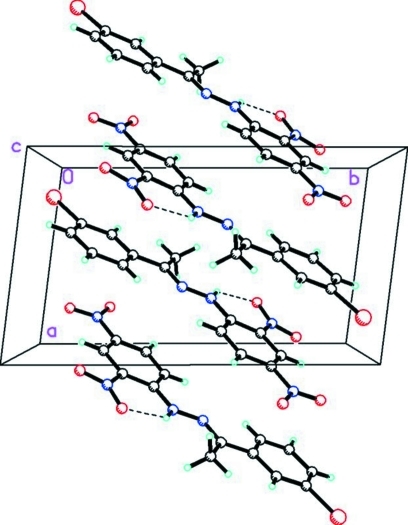
Packing diagram of C14H11N4O4Br viewed down the c axis.

## supplementary crystallographic information

### Comment

Schiff bases and their complexes are widely used in the fields of biology and catalysis (Liang, 2007). In particular the dinitrophenyl hydrazones exhibit good nonlinear optical (NLO) and crystalline properties (Baughman *et al.*, 2004) and are found to have versatile coordinating abilities towards different metal ions. In addition, some 2,4-dinitrophenyl hydrazone derivatives have been shown to be potentially DNA-damaging and mutagenic agents (Okabe *et al.*, 1993). As a result of their significant molecular nonlinearities and remarkable ability to crystallize in non-centrosymmetric crystal systems (Zare *et al.*, 2005; El-Seify *et al.*, 2006) many X-ray structural studies of 2,4-dinitrophenylhydrazones have been reported. Among them, the most closely related structures are (*E*)-*p*-methoxy-acetophenone 2,4-dinitrophenylhydrazone (Bolte & Dill, 1998), acetophenone (2,4-dinitrophenyl)hydrazone (Shan *et al.*, 2002), 3-chloroacetophenone 2,4-dintrophenyl-hydrazone (Fan *et al.*, 2004), 2,4-dihydroxyacetophenone 2,4-dinitrophenylhydrazone (Baughman *et al.*, 2004), *syn*-acetophenone (2,4-dinitrophenyl)hydrazone (Motherwell & Ramsay, 2007), *N*-(2,4-dinitrophenyl)-*N*'-(1-*p*-tolylethylidene)hydrazine (Kia *et al.*, 2009) and *N*-(2,4-dinitro-phenyl)-*N*'-(1-phenyl-ethylidene)- hydrazine (Ji *et al.*, 2010). In view of the importance of 2,4-dinitrophenylhydrazones, this paper reports the crystal structure of C_14_H_11_N_4_O_4_Br.

The title compound, C_14_H_11_N_4_O_4_Br, contains 3-bromophenyl and 2,4-dinitrophenyl groups on opposite sides of a hydrazone moiety. Two molecules (A & B) are present in the asymmetric unit (Fig. 1). The Br atom in molecule B is disordered across the *meta* positions of the benzene ring (Br2B and Br2C occupancies of 0.873 (1) and 0.127 (1), respectively). The dihedral angles between the least squares planes of the two benzene rings in each structure are 2.01° (A) and 2.58° (B), respectively. Weak C—H···O hydrogen bonds (Table 1) and weak π···π stacking interactions [*Cg*1···*Cg*2^i^ and *Cg*1···*Cg*3^i^ = 3.7269 (14)Å and where *Cg*1, *Cg*2, *Cg*3 = centroids for C1B···C6B, C9B—C14B and C9B/C10B/C11C/C12B/C13C/C14B, respectively; ^i^ = 1 - *x*, 1 - *y*, 1 - *x*] contribute to the stability of the crystal packing (Fig.2). In addition there are N—H···O hydrogen bonds involving the N-H and adjacent nitro O atoms.

### Experimental

A mixture of 2,4-dinitrophenylhydrazine (1.98 g) and 1-(3-bromophenyl)ethanone (1.99 g) was dissolved in methanol and refluxed for about 6 h (Fig. 3). The precipitate formed was filtered, dried and recrystallized in ethyl acetate. X-ray quality crystals of the title compound were obtained after three days by the slow evaporation of ethyl acetate solution at room temperature. (mp: 497- 499 K).

### Refinement

All of the H atoms were placed in their calculated positions and then refined using the riding model with Atom—H lengths of 0.93Å (CH), 0.96Å (CH_3_) or 0.86Å (NH). Isotropic displacement parameters for these atoms were set to 1.2 times (NH), 1.2 (CH) or 1.5 (CH_3_) times *U*_eq_ of the parent atom. For one of the rings the the Br is disordered over two equivalent positions with occupancies of 0.873 (1) and 0.127 (1).

### Crystal data

**Table:**

C_14_H_11_BrN_4_O_4_	*Z* = 4
*M**_r_* = 379.18	*F*(000) = 760
Triclinic, *P*1	*D*_x_ = 1.718 Mg m^−^^3^
Hall symbol: -P 1	Mo *K*α radiation, λ = 0.71073 Å
*a* = 7.7546 (9) Å	Cell parameters from 5432 reflections
*b* = 13.4362 (15) Å	θ = 2.7–30.8°
*c* = 14.1884 (16) Å	µ = 2.83 mm^−^^1^
α = 91.894 (2)°	*T* = 100 K
β = 90.553 (2)°	Block, orange
γ = 97.128 (2)°	0.55 × 0.55 × 0.24 mm
*V* = 1466.0 (3) Å^3^	

### Data collection

**Table:**

Bruker APEXII CCD diffractometer	8581 independent reflections
Radiation source: fine-focus sealed tube	6680 reflections with *I* > 2σ(*I*)
graphite	*R*_int_ = 0.024
ω scans	θ_max_ = 31.3°, θ_min_ = 1.4°
Absorption correction: multi-scan (*SADABS*; Bruker, 2008)	*h* = −11→11
*T*_min_ = 0.547, *T*_max_ = 0.746	*k* = −19→19
16795 measured reflections	*l* = −20→19

### Refinement

**Table:**

Refinement on *F*^2^	Primary atom site location: structure-invariant direct methods
Least-squares matrix: full	Secondary atom site location: difference Fourier map
*R*[*F*^2^ > 2σ(*F*^2^)] = 0.040	Hydrogen site location: inferred from neighbouring sites
*wR*(*F*^2^) = 0.102	H-atom parameters constrained
*S* = 1.03	*w* = 1/[σ^2^(*F*_o_^2^) + (0.049*P*)^2^ + 0.9159*P*] where *P* = (*F*_o_^2^ + 2*F*_c_^2^)/3
8581 reflections	(Δ/σ)_max_ = 0.002
428 parameters	Δρ_max_ = 1.44 e Å^−^^3^
2 restraints	Δρ_min_ = −0.77 e Å^−^^3^

### Special details

**Table:**

Experimental. 2010-03-28 # Formatted by publCIF 2010-08-17 # Formatted by IUCr publCIF system
Geometry. All esds (except the esd in the dihedral angle between two l.s. planes) are estimated using the full covariance matrix. The cell esds are taken into account individually in the estimation of esds in distances, angles and torsion angles; correlations between esds in cell parameters are only used when they are defined by crystal symmetry. An approximate (isotropic) treatment of cell esds is used for estimating esds involving l.s. planes.
Refinement. Refinement of F^2^ against ALL reflections. The weighted R-factor wR and goodness of fit S are based on F^2^, conventional R-factors R are based on F, with F set to zero for negative F^2^. The threshold expression of F^2^ > 2sigma(F^2^) is used only for calculating R-factors(gt) etc. and is not relevant to the choice of reflections for refinement. R-factors based on F^2^ are statistically about twice as large as those based on F, and R- factors based on ALL data will be even larger.

### Fractional atomic coordinates and isotropic or equivalent isotropic displacement parameters (Å^2^)

**Table:**

	*x*	*y*	*z*	*U*_iso_*/*U*_eq_	Occ. (<1)
C11B	0.3320 (3)	0.11977 (16)	0.42870 (18)	0.0279 (5)	0.8734 (10)
C13B	0.4862 (3)	0.17310 (18)	0.56961 (15)	0.0281 (5)	0.8734 (10)
H13B	0.5206	0.1596	0.6319	0.034*	0.8734 (10)
C11C	0.3320 (3)	0.11977 (16)	0.42870 (18)	0.0279 (5)	0.1266 (10)
H11C	0.2387	0.0610	0.3838	0.034*	0.1266 (10)
C13C	0.4862 (3)	0.17310 (18)	0.56961 (15)	0.0281 (5)	0.1266 (10)
Br2B	0.19142 (4)	0.02208 (2)	0.36023 (3)	0.04126 (11)	0.8734 (10)
Br2C	0.5634 (3)	0.13884 (16)	0.68483 (14)	0.0364 (7)	0.1266 (10)
O1B	0.7504 (2)	0.67942 (12)	0.30264 (11)	0.0236 (3)	
O2B	0.9336 (2)	0.81206 (12)	0.32857 (12)	0.0291 (4)	
O3B	1.2253 (2)	0.88614 (12)	0.61872 (12)	0.0276 (4)	
O4B	1.2248 (2)	0.77225 (13)	0.72350 (11)	0.0265 (3)	
N1B	0.7215 (2)	0.53546 (13)	0.42320 (12)	0.0171 (3)	
H1B	0.6906	0.5510	0.3677	0.021*	
N2B	0.6603 (2)	0.44461 (13)	0.45865 (13)	0.0178 (3)	
N3B	0.8600 (2)	0.73102 (13)	0.35350 (12)	0.0196 (4)	
N4B	1.1765 (2)	0.80244 (14)	0.64777 (13)	0.0198 (4)	
C1B	0.8319 (3)	0.60103 (15)	0.47668 (14)	0.0159 (4)	
C2B	0.9025 (3)	0.69629 (15)	0.44510 (14)	0.0161 (4)	
C3B	1.0139 (3)	0.76242 (15)	0.50129 (15)	0.0172 (4)	
H3B	1.0591	0.8263	0.4793	0.021*	
C4B	1.0576 (3)	0.73365 (15)	0.58945 (15)	0.0175 (4)	
C5B	0.9929 (3)	0.64026 (15)	0.62373 (15)	0.0180 (4)	
H5B	1.0254	0.6220	0.6849	0.022*	
C6B	0.8825 (3)	0.57554 (15)	0.56820 (14)	0.0171 (4)	
H6B	0.8386	0.5120	0.5915	0.021*	
C7B	0.5594 (3)	0.38472 (15)	0.40366 (15)	0.0176 (4)	
C8B	0.5111 (3)	0.41146 (17)	0.30597 (16)	0.0245 (5)	
H6	0.4551	0.4730	0.3090	0.037*	
H7	0.4306	0.3566	0.2772	0.037*	
H8	0.6161	0.4223	0.2679	0.037*	
C9B	0.4959 (3)	0.28630 (15)	0.44326 (16)	0.0189 (4)	
C10B	0.3893 (3)	0.21275 (16)	0.39073 (17)	0.0225 (4)	
H10B	0.3554	0.2248	0.3295	0.027*	
C12B	0.3816 (3)	0.10017 (17)	0.52082 (19)	0.0291 (5)	
H12B	0.3434	0.0379	0.5482	0.035*	
C14B	0.5455 (3)	0.26506 (16)	0.53498 (16)	0.0226 (4)	
H14B	0.6185	0.3133	0.5724	0.027*	
Br1A	0.46515 (3)	0.585783 (17)	0.117323 (18)	0.02724 (7)	
O1A	1.1072 (2)	1.23013 (12)	0.17885 (11)	0.0250 (3)	
O2A	1.2017 (2)	1.38349 (12)	0.14856 (12)	0.0279 (4)	
O3A	1.1250 (2)	1.52151 (13)	−0.14936 (13)	0.0300 (4)	
O4A	0.9604 (3)	1.43670 (13)	−0.25602 (12)	0.0320 (4)	
N1A	0.9017 (2)	1.11916 (13)	0.05847 (13)	0.0191 (3)	
H1A	0.9406	1.1219	0.1156	0.023*	
N2A	0.8042 (2)	1.03459 (13)	0.02230 (13)	0.0199 (4)	
N3A	1.1202 (2)	1.30168 (14)	0.12536 (13)	0.0200 (4)	
N4A	1.0306 (3)	1.44583 (15)	−0.17722 (14)	0.0241 (4)	
C1A	0.9364 (3)	1.19896 (16)	0.00283 (15)	0.0185 (4)	
C2A	1.0392 (3)	1.28937 (16)	0.03282 (15)	0.0179 (4)	
C3A	1.0704 (3)	1.37046 (16)	−0.02584 (16)	0.0201 (4)	
H3A	1.1401	1.4304	−0.0047	0.024*	
C4A	0.9983 (3)	1.36193 (16)	−0.11485 (16)	0.0210 (4)	
C5A	0.8957 (3)	1.27486 (17)	−0.14749 (16)	0.0233 (4)	
H5A	0.8470	1.2710	−0.2094	0.028*	
C6A	0.8655 (3)	1.19486 (17)	−0.09004 (15)	0.0223 (4)	
H6A	0.7960	1.1356	−0.1127	0.027*	
C7A	0.7589 (3)	0.96430 (16)	0.08055 (15)	0.0204 (4)	
C8A	0.8080 (5)	0.9730 (2)	0.18295 (19)	0.0453 (8)	
H8A	0.7673	1.0332	0.2114	0.068*	
H8B	0.9347	0.9780	0.1898	0.068*	
H8C	0.7543	0.9136	0.2148	0.068*	
C9A	0.6534 (3)	0.87396 (16)	0.03918 (15)	0.0183 (4)	
C10A	0.6164 (3)	0.78681 (16)	0.09002 (15)	0.0200 (4)	
H10A	0.6583	0.7845	0.1530	0.024*	
C11A	0.5178 (3)	0.70347 (16)	0.04771 (16)	0.0204 (4)	
C12A	0.4544 (3)	0.70369 (17)	−0.04363 (16)	0.0237 (4)	
H12A	0.3871	0.6460	−0.0713	0.028*	
C13A	0.4918 (3)	0.79094 (17)	−0.09411 (16)	0.0232 (4)	
H13A	0.4496	0.7928	−0.1570	0.028*	
C14A	0.5887 (3)	0.87411 (17)	−0.05387 (15)	0.0212 (4)	
H14A	0.6127	0.9328	−0.0894	0.025*	

### Atomic displacement parameters (Å^2^)

**Table:**

	*U*^11^	*U*^22^	*U*^33^	*U*^12^	*U*^13^	*U*^23^
C11B	0.0214 (11)	0.0159 (10)	0.0454 (15)	−0.0015 (8)	−0.0004 (10)	−0.0014 (9)
C13B	0.0296 (12)	0.0218 (11)	0.0332 (13)	0.0029 (9)	0.0039 (10)	0.0080 (9)
C11C	0.0214 (11)	0.0159 (10)	0.0454 (15)	−0.0015 (8)	−0.0004 (10)	−0.0014 (9)
C13C	0.0296 (12)	0.0218 (11)	0.0332 (13)	0.0029 (9)	0.0039 (10)	0.0080 (9)
Br2B	0.03745 (18)	0.01748 (14)	0.0650 (2)	−0.00971 (11)	−0.01999 (15)	−0.00010 (13)
Br2C	0.0462 (13)	0.0301 (11)	0.0300 (11)	−0.0101 (8)	−0.0098 (9)	0.0181 (8)
O1B	0.0299 (8)	0.0207 (8)	0.0189 (7)	−0.0016 (6)	−0.0053 (6)	0.0013 (6)
O2B	0.0428 (10)	0.0198 (8)	0.0217 (8)	−0.0101 (7)	−0.0024 (7)	0.0078 (6)
O3B	0.0326 (9)	0.0194 (8)	0.0283 (9)	−0.0068 (6)	−0.0036 (7)	−0.0008 (6)
O4B	0.0268 (8)	0.0307 (9)	0.0207 (8)	−0.0016 (7)	−0.0068 (6)	0.0010 (7)
N1B	0.0200 (8)	0.0139 (8)	0.0165 (8)	−0.0017 (6)	−0.0017 (7)	0.0031 (6)
N2B	0.0185 (8)	0.0137 (8)	0.0205 (9)	−0.0011 (6)	0.0012 (7)	0.0023 (6)
N3B	0.0252 (9)	0.0164 (8)	0.0167 (8)	0.0007 (7)	0.0006 (7)	0.0010 (7)
N4B	0.0167 (8)	0.0199 (9)	0.0217 (9)	−0.0012 (6)	−0.0006 (7)	−0.0029 (7)
C1B	0.0162 (9)	0.0152 (9)	0.0159 (9)	0.0004 (7)	0.0021 (7)	0.0004 (7)
C2B	0.0191 (9)	0.0149 (9)	0.0140 (9)	0.0001 (7)	−0.0004 (7)	0.0025 (7)
C3B	0.0174 (9)	0.0140 (9)	0.0197 (10)	−0.0005 (7)	0.0021 (7)	0.0005 (7)
C4B	0.0160 (9)	0.0167 (9)	0.0187 (10)	−0.0012 (7)	−0.0006 (7)	−0.0017 (7)
C5B	0.0189 (9)	0.0177 (9)	0.0173 (9)	0.0015 (7)	0.0000 (7)	0.0023 (7)
C6B	0.0188 (9)	0.0146 (9)	0.0176 (9)	0.0004 (7)	0.0003 (7)	0.0021 (7)
C7B	0.0166 (9)	0.0153 (9)	0.0204 (10)	0.0001 (7)	0.0007 (7)	0.0020 (7)
C8B	0.0285 (11)	0.0208 (11)	0.0221 (11)	−0.0059 (8)	−0.0054 (9)	0.0047 (8)
C9B	0.0160 (9)	0.0152 (9)	0.0255 (11)	0.0012 (7)	0.0027 (8)	0.0009 (8)
C10B	0.0186 (10)	0.0168 (10)	0.0314 (12)	0.0001 (8)	−0.0006 (8)	−0.0012 (8)
C12B	0.0275 (12)	0.0163 (10)	0.0437 (15)	0.0014 (8)	0.0060 (10)	0.0070 (10)
C14B	0.0231 (10)	0.0182 (10)	0.0260 (11)	−0.0001 (8)	0.0016 (9)	0.0017 (8)
Br1A	0.02909 (13)	0.01787 (11)	0.03320 (13)	−0.00395 (8)	−0.00194 (9)	0.00412 (9)
O1A	0.0301 (9)	0.0221 (8)	0.0220 (8)	−0.0007 (6)	−0.0029 (6)	0.0035 (6)
O2A	0.0280 (9)	0.0207 (8)	0.0326 (9)	−0.0055 (6)	−0.0078 (7)	−0.0011 (7)
O3A	0.0301 (9)	0.0219 (8)	0.0375 (10)	−0.0004 (7)	0.0038 (7)	0.0078 (7)
O4A	0.0447 (11)	0.0289 (9)	0.0232 (9)	0.0065 (8)	0.0014 (8)	0.0064 (7)
N1A	0.0214 (9)	0.0170 (8)	0.0177 (8)	−0.0024 (7)	−0.0026 (7)	−0.0003 (7)
N2A	0.0196 (9)	0.0176 (8)	0.0213 (9)	−0.0016 (7)	−0.0006 (7)	−0.0016 (7)
N3A	0.0177 (8)	0.0205 (9)	0.0215 (9)	0.0016 (7)	−0.0003 (7)	−0.0005 (7)
N4A	0.0260 (10)	0.0228 (9)	0.0247 (10)	0.0061 (7)	0.0072 (8)	0.0046 (8)
C1A	0.0183 (9)	0.0169 (9)	0.0201 (10)	0.0016 (7)	0.0033 (8)	0.0003 (8)
C2A	0.0174 (9)	0.0188 (10)	0.0174 (10)	0.0020 (7)	−0.0001 (7)	−0.0007 (7)
C3A	0.0186 (10)	0.0174 (10)	0.0243 (11)	0.0022 (7)	0.0037 (8)	0.0000 (8)
C4A	0.0216 (10)	0.0194 (10)	0.0227 (11)	0.0034 (8)	0.0049 (8)	0.0052 (8)
C5A	0.0254 (11)	0.0249 (11)	0.0195 (10)	0.0026 (8)	0.0014 (8)	0.0007 (8)
C6A	0.0246 (11)	0.0208 (10)	0.0205 (10)	−0.0010 (8)	0.0005 (8)	−0.0008 (8)
C7A	0.0245 (11)	0.0171 (10)	0.0194 (10)	0.0025 (8)	−0.0028 (8)	−0.0003 (8)
C8A	0.088 (2)	0.0196 (12)	0.0236 (13)	−0.0116 (13)	−0.0209 (14)	0.0033 (10)
C9A	0.0182 (9)	0.0169 (9)	0.0198 (10)	0.0025 (7)	−0.0006 (8)	−0.0014 (8)
C10A	0.0206 (10)	0.0194 (10)	0.0197 (10)	0.0016 (8)	−0.0021 (8)	0.0008 (8)
C11A	0.0193 (10)	0.0170 (10)	0.0245 (11)	0.0006 (7)	0.0013 (8)	0.0006 (8)
C12A	0.0203 (10)	0.0230 (11)	0.0268 (11)	0.0001 (8)	−0.0018 (8)	−0.0038 (9)
C13A	0.0235 (11)	0.0279 (11)	0.0175 (10)	0.0016 (8)	−0.0040 (8)	−0.0023 (8)
C14A	0.0248 (10)	0.0203 (10)	0.0185 (10)	0.0024 (8)	−0.0012 (8)	0.0016 (8)

### Geometric parameters (Å, °)

**Table:**

C11B—C10B	1.400 (3)	Br1A—C11A	1.896 (2)
C11B—C12B	1.401 (4)	O1A—N3A	1.239 (2)
C11B—Br2B	1.843 (2)	O2A—N3A	1.231 (2)
C11B—H11C	1.1703	O3A—N4A	1.229 (3)
C13B—C12B	1.358 (3)	O4A—N4A	1.234 (3)
C13B—C14B	1.372 (3)	N1A—C1A	1.354 (3)
C13B—H13B	0.9500	N1A—N2A	1.366 (2)
O1B—N3B	1.238 (2)	N1A—H1A	0.8599
O2B—N3B	1.229 (2)	N2A—C7A	1.294 (3)
O3B—N4B	1.227 (2)	N3A—C2A	1.446 (3)
O4B—N4B	1.232 (2)	N4A—C4A	1.455 (3)
N1B—C1B	1.357 (3)	C1A—C2A	1.418 (3)
N1B—N2B	1.367 (2)	C1A—C6A	1.419 (3)
N1B—H1B	0.8604	C2A—C3A	1.392 (3)
N2B—C7B	1.288 (3)	C3A—C4A	1.372 (3)
N3B—C2B	1.444 (3)	C3A—H3A	0.9500
N4B—C4B	1.454 (3)	C4A—C5A	1.394 (3)
C1B—C2B	1.417 (3)	C5A—C6A	1.369 (3)
C1B—C6B	1.418 (3)	C5A—H5A	0.9500
C2B—C3B	1.386 (3)	C6A—H6A	0.9500
C3B—C4B	1.373 (3)	C7A—C9A	1.478 (3)
C3B—H3B	0.9500	C7A—C8A	1.496 (3)
C4B—C5B	1.397 (3)	C8A—H8A	0.9800
C5B—C6B	1.366 (3)	C8A—H8B	0.9800
C5B—H5B	0.9500	C8A—H8C	0.9800
C6B—H6B	0.9500	C9A—C10A	1.396 (3)
C7B—C9B	1.483 (3)	C9A—C14A	1.408 (3)
C7B—C8B	1.499 (3)	C10A—C11A	1.390 (3)
C8B—H6	0.9800	C10A—H10A	0.9500
C8B—H7	0.9800	C11A—C12A	1.382 (3)
C8B—H8	0.9800	C12A—C13A	1.395 (3)
C9B—C10B	1.397 (3)	C12A—H12A	0.9500
C9B—C14B	1.402 (3)	C13A—C14A	1.372 (3)
C10B—H10B	0.9299	C13A—H13A	0.9500
C12B—H12B	0.9500	C14A—H14A	0.9500
C14B—H14B	0.9500		
			
C10B—C11B—C12B	119.6 (2)	C9B—C14B—H14B	120.7
C10B—C11B—Br2B	121.40 (19)	C1A—N1A—N2A	118.89 (18)
C12B—C11B—Br2B	118.98 (17)	C1A—N1A—H1A	120.3
C10B—C11B—H11C	119.1	N2A—N1A—H1A	120.8
C12B—C11B—H11C	121.2	C7A—N2A—N1A	116.76 (18)
C12B—C13B—C14B	124.4 (2)	O2A—N3A—O1A	121.74 (19)
C12B—C13B—H13B	117.8	O2A—N3A—C2A	118.64 (18)
C14B—C13B—H13B	117.8	O1A—N3A—C2A	119.62 (18)
C1B—N1B—N2B	119.14 (17)	O3A—N4A—O4A	123.5 (2)
C1B—N1B—H1B	120.2	O3A—N4A—C4A	119.0 (2)
N2B—N1B—H1B	120.6	O4A—N4A—C4A	117.6 (2)
C7B—N2B—N1B	116.20 (18)	N1A—C1A—C2A	123.4 (2)
O2B—N3B—O1B	121.78 (18)	N1A—C1A—C6A	119.72 (19)
O2B—N3B—C2B	118.60 (18)	C2A—C1A—C6A	116.92 (19)
O1B—N3B—C2B	119.62 (17)	C3A—C2A—C1A	121.85 (19)
O3B—N4B—O4B	123.68 (18)	C3A—C2A—N3A	116.29 (19)
O3B—N4B—C4B	118.75 (18)	C1A—C2A—N3A	121.84 (18)
O4B—N4B—C4B	117.56 (18)	C4A—C3A—C2A	118.5 (2)
N1B—C1B—C2B	122.79 (18)	C4A—C3A—H3A	120.8
N1B—C1B—C6B	120.28 (18)	C2A—C3A—H3A	120.8
C2B—C1B—C6B	116.93 (18)	C3A—C4A—C5A	121.8 (2)
C3B—C2B—C1B	121.79 (18)	C3A—C4A—N4A	119.2 (2)
C3B—C2B—N3B	116.10 (17)	C5A—C4A—N4A	119.0 (2)
C1B—C2B—N3B	122.10 (18)	C6A—C5A—C4A	119.8 (2)
C4B—C3B—C2B	118.59 (18)	C6A—C5A—H5A	120.1
C4B—C3B—H3B	120.7	C4A—C5A—H5A	120.1
C2B—C3B—H3B	120.7	C5A—C6A—C1A	121.1 (2)
C3B—C4B—C5B	121.95 (19)	C5A—C6A—H6A	119.4
C3B—C4B—N4B	118.55 (18)	C1A—C6A—H6A	119.4
C5B—C4B—N4B	119.49 (19)	N2A—C7A—C9A	115.38 (19)
C6B—C5B—C4B	119.20 (19)	N2A—C7A—C8A	122.9 (2)
C6B—C5B—H5B	120.4	C9A—C7A—C8A	121.8 (2)
C4B—C5B—H5B	120.4	C7A—C8A—H8A	109.5
C5B—C6B—C1B	121.53 (19)	C7A—C8A—H8B	109.5
C5B—C6B—H6B	119.2	H8A—C8A—H8B	109.5
C1B—C6B—H6B	119.2	C7A—C8A—H8C	109.5
N2B—C7B—C9B	115.24 (19)	H8A—C8A—H8C	109.5
N2B—C7B—C8B	122.65 (19)	H8B—C8A—H8C	109.5
C9B—C7B—C8B	122.10 (19)	C10A—C9A—C14A	118.40 (19)
C7B—C8B—H6	109.5	C10A—C9A—C7A	121.57 (19)
C7B—C8B—H7	109.5	C14A—C9A—C7A	120.02 (19)
H6—C8B—H7	109.5	C11A—C10A—C9A	119.4 (2)
C7B—C8B—H8	109.5	C11A—C10A—H10A	120.3
H6—C8B—H8	109.5	C9A—C10A—H10A	120.3
H7—C8B—H8	109.5	C12A—C11A—C10A	122.2 (2)
C10B—C9B—C14B	118.6 (2)	C12A—C11A—Br1A	118.45 (16)
C10B—C9B—C7B	121.6 (2)	C10A—C11A—Br1A	119.39 (17)
C14B—C9B—C7B	119.75 (19)	C11A—C12A—C13A	118.2 (2)
C9B—C10B—C11B	120.9 (2)	C11A—C12A—H12A	120.9
C9B—C10B—H10B	120.3	C13A—C12A—H12A	120.9
C11B—C10B—H10B	118.8	C14A—C13A—C12A	120.7 (2)
C13B—C12B—C11B	117.9 (2)	C14A—C13A—H13A	119.6
C13B—C12B—H12B	121.1	C12A—C13A—H13A	119.6
C11B—C12B—H12B	121.1	C13A—C14A—C9A	121.1 (2)
C13B—C14B—C9B	118.6 (2)	C13A—C14A—H14A	119.4
C13B—C14B—H14B	120.7	C9A—C14A—H14A	119.4
			
C1B—N1B—N2B—C7B	−178.38 (18)	C1A—N1A—N2A—C7A	−172.92 (19)
N2B—N1B—C1B—C2B	179.47 (18)	N2A—N1A—C1A—C2A	−178.63 (19)
N2B—N1B—C1B—C6B	−0.2 (3)	N2A—N1A—C1A—C6A	2.5 (3)
N1B—C1B—C2B—C3B	179.45 (19)	N1A—C1A—C2A—C3A	−179.2 (2)
C6B—C1B—C2B—C3B	−0.9 (3)	C6A—C1A—C2A—C3A	−0.3 (3)
N1B—C1B—C2B—N3B	0.7 (3)	N1A—C1A—C2A—N3A	2.1 (3)
C6B—C1B—C2B—N3B	−179.63 (18)	C6A—C1A—C2A—N3A	−179.05 (19)
O2B—N3B—C2B—C3B	5.3 (3)	O2A—N3A—C2A—C3A	4.2 (3)
O1B—N3B—C2B—C3B	−174.14 (19)	O1A—N3A—C2A—C3A	−175.28 (19)
O2B—N3B—C2B—C1B	−175.93 (19)	O2A—N3A—C2A—C1A	−176.97 (19)
O1B—N3B—C2B—C1B	4.6 (3)	O1A—N3A—C2A—C1A	3.5 (3)
C1B—C2B—C3B—C4B	0.7 (3)	C1A—C2A—C3A—C4A	0.3 (3)
N3B—C2B—C3B—C4B	179.46 (18)	N3A—C2A—C3A—C4A	179.14 (19)
C2B—C3B—C4B—C5B	−0.1 (3)	C2A—C3A—C4A—C5A	0.0 (3)
C2B—C3B—C4B—N4B	179.09 (18)	C2A—C3A—C4A—N4A	−179.65 (19)
O3B—N4B—C4B—C3B	4.4 (3)	O3A—N4A—C4A—C3A	1.7 (3)
O4B—N4B—C4B—C3B	−174.42 (19)	O4A—N4A—C4A—C3A	−178.3 (2)
O3B—N4B—C4B—C5B	−176.35 (19)	O3A—N4A—C4A—C5A	−178.0 (2)
O4B—N4B—C4B—C5B	4.8 (3)	O4A—N4A—C4A—C5A	2.1 (3)
C3B—C4B—C5B—C6B	−0.2 (3)	C3A—C4A—C5A—C6A	−0.3 (3)
N4B—C4B—C5B—C6B	−179.38 (18)	N4A—C4A—C5A—C6A	179.3 (2)
C4B—C5B—C6B—C1B	−0.1 (3)	C4A—C5A—C6A—C1A	0.3 (3)
N1B—C1B—C6B—C5B	−179.74 (19)	N1A—C1A—C6A—C5A	178.9 (2)
C2B—C1B—C6B—C5B	0.6 (3)	C2A—C1A—C6A—C5A	0.0 (3)
N1B—N2B—C7B—C9B	179.45 (17)	N1A—N2A—C7A—C9A	179.93 (18)
N1B—N2B—C7B—C8B	0.4 (3)	N1A—N2A—C7A—C8A	0.2 (3)
N2B—C7B—C9B—C10B	−178.26 (19)	N2A—C7A—C9A—C10A	170.6 (2)
C8B—C7B—C9B—C10B	0.8 (3)	C8A—C7A—C9A—C10A	−9.7 (4)
N2B—C7B—C9B—C14B	0.7 (3)	N2A—C7A—C9A—C14A	−8.8 (3)
C8B—C7B—C9B—C14B	179.8 (2)	C8A—C7A—C9A—C14A	170.9 (2)
C14B—C9B—C10B—C11B	0.3 (3)	C14A—C9A—C10A—C11A	0.0 (3)
C7B—C9B—C10B—C11B	179.3 (2)	C7A—C9A—C10A—C11A	−179.5 (2)
C12B—C11B—C10B—C9B	0.3 (3)	C9A—C10A—C11A—C12A	0.0 (3)
Br2B—C11B—C10B—C9B	−179.97 (17)	C9A—C10A—C11A—Br1A	−179.19 (16)
C14B—C13B—C12B—C11B	0.5 (4)	C10A—C11A—C12A—C13A	0.1 (3)
C10B—C11B—C12B—C13B	−0.6 (4)	Br1A—C11A—C12A—C13A	179.22 (17)
Br2B—C11B—C12B—C13B	179.58 (18)	C11A—C12A—C13A—C14A	−0.1 (3)
C12B—C13B—C14B—C9B	0.0 (4)	C12A—C13A—C14A—C9A	0.1 (3)
C10B—C9B—C14B—C13B	−0.4 (3)	C10A—C9A—C14A—C13A	0.0 (3)
C7B—C9B—C14B—C13B	−179.4 (2)	C7A—C9A—C14A—C13A	179.5 (2)

### Hydrogen-bond geometry (Å, °)

**Table:**

*D*—H···*A*	*D*—H	H···*A*	*D*···*A*	*D*—H···*A*
N1B—H1B···O1B	0.86	1.99	2.615 (2)	129
N1A—H1A···O1A	0.86	2.00	2.621 (2)	128
C8B—H7···O2A^i^	0.98	2.60	3.241 (3)	123
C12B—H12B···O3B^i^	0.95	2.38	3.332 (3)	175
C8A—H8A···O3B^ii^	0.98	2.61	3.369 (3)	135
C13A—H13A···O4B^iii^	0.95	2.40	3.282 (3)	154

Symmetry codes: (i) *x*−1, *y*−1, *z*; (ii) −*x*+2, −*y*+2, −*z*+1; (iii) *x*−1, *y*, *z*−1.
